# Genomics of Cancer and a New Era for Cancer Prevention

**DOI:** 10.1371/journal.pgen.1005522

**Published:** 2015-11-05

**Authors:** Paul Brennan, Christopher P. Wild

**Affiliations:** 1 Section of Genetics, International Agency for Research on Cancer, Lyon, France; 2 Director’s office, International Agency for Research on Cancer, Lyon, France; National Cancer Institute, UNITED STATES

## Abstract

A primary justification for dedicating substantial amounts of research funding to large-scale cancer genomics projects of both somatic and germline DNA is that the biological insights will lead to new treatment targets and strategies for cancer therapy. While it is too early to judge the success of these projects in terms of clinical breakthroughs, an alternative rationale is that new genomics techniques can be used to reduce the overall burden of cancer by prevention of new cases occurring and also by detecting them earlier. In particular, it is now becoming apparent that studying the genomic profile of tumors can help to identify new carcinogens and may subsequently result in implementing strategies that limit exposure. In parallel, it may be feasible to utilize genomic biomarkers to identify cancers at an earlier and more treatable stage using screening or other early detection approaches based on prediagnostic biospecimens. While the potential for these techniques is large, their successful outcome will depend on international collaboration and planning similar to that of recent sequencing initiatives.

Since the publication of the initial human genome sequence in 2002, at a cost of around US$3 thousand million, DNA sequencing has advanced to the extent where whole genomes can be sequenced in days for around one millionth of the cost [1]. This has led to a scientific tour de force in projects that aim to understand the genetics of cancer. Large-scale initiatives such as the International Cancer Genome Consortium (ICGC) and the Cancer Genome Atlas (TCGA) for somatic variation, as well as the OncoArray Network for genome-wide studies of germline variation, have harnessed international expertise in oncology, genomics, and bioinformatics with very high levels of funding and have resulted in the coordinated genotyping, sequencing, and cataloging of many thousands of cancer cases [2]. Comprehensive genomic data from all completed cases are being made available to the research community, along with basic clinical information on some, allowing for extensive additional analyses. This initiative has led to a new understanding of how to define specific cancer subtypes and has vastly increased the pace of progress in elucidating the underlying biology of cancer [3].

The most prominent visible outcome of the increased understanding of cancer biology is that targeted treatments have been developed or are being tested that aim to block specific molecules that spur the growth or spread of cancer. Although there are some exciting success stories such as the vastly improved survival with imatinib and chronic myelogenous leukemia (CML) or the increased efficacy of Herceptin treatment for women with Her2-positive breast cancer, most of this new generation of targeted treatments promise, at most, only a partial respite from the disease. The typical scenario is that the underlying cancer is not totally eradicated, remnants of the disease evolve and overcome any treatment, and the relapse is severe [4].

New targeted therapies are also expensive to develop and to prescribe, some costing over US$100,000 for each patient per year, while being applicable for a smaller number of patients with the relevant subtype of disease. Disease resistance may be overcome through new strategies that combine therapies for specific pathways, and combination therapy of two or more drugs that target independent pathways is likely to hold even greater promise for improving response [5]. Other approaches such as combined use of immune checkpoint inhibitors are also providing exciting results [6], although there remain concerns that the strategy of developing targeted therapies for late-stage disease may be fundamentally flawed, given the inherent complexity and heterogeneity of such tumors [7,8]. A complementary approach would be to focus also on early detection of localized cancer, including the use of screening, when survival is usually a lot more favorable [3], as well as primary prevention in identifying the causes and minimizing exposure. The role of genomics in primary and secondary prevention of cancer has received less attention than treatment, although it is perhaps here that genomics will have its most important contribution in the long term.

## Primary Prevention of Cancer—Stopping the Disease Occurring

Some of the greatest public health successes in cancer prevention have arisen from identifying the causes of cancer and limiting or removing the exposure [9]. Obvious examples include identifying the role of smoking for lung cancer [10], and later for another 17 cancer types [11], implementation of Hepatitis-B vaccination programs against liver cancer [12], the role of Human Papilloma virus (HPV) in cervical cancer that led directly to the development of prophylactic vaccines [13], and the identification of specific occupations associated with very high cancer risk that has resulted in subsequent control of these exposures in many, but not all, parts of the world (e.g., workers exposed to asbestos and risk of mesothelioma). Overall, about 40% of cancer cases in high income countries appear to be attributable to known lifestyle factors, with tobacco explaining about half of this amount [14,15], indicating that much remains to be done in limiting the effects of this exposure. Such is the role of tobacco, that it can be useful to consider the proportion of cancers avoidable by known risk factors in smokers and nonsmokers separately. In an exercise for cancer deaths in the United States, about 70% of cancer deaths among smokers could be accounted for by known causes (60% due to tobacco and 10% due to other exposures), whereas only about 20% of cancer deaths among nonsmokers could be attributable to known causes [14].

There clearly remain important gaps in the litany of what causes cancers to occur, especially among the majority of the population who do not smoke. While some cancers are relatively rare in all populations, it is also the case that all cancers that are common in some populations are much rarer in others, usually by an order of magnitude or more [16]. It is clear from migrant studies and time trends that genetic susceptibility cannot explain these differences, implying underlying lifestyle and environmental factors. High quality cancer registries around the world are capable of accurately recording the numbers of new cases of each cancer type in a population, allowing for valid international comparisons [16]. The seven cancers listed in Table 1 make up over 25% of the global cancer burden, although only a small part of these international differences can currently be explained [16]. The incidence of some cancers has increased rapidly in recent years in different regions of the world, including testicular and renal cancers, as well as lymphomas. Despite multiple efforts, traditional epidemiology studies have not been able to explain these increases.

**Table 1:** Cancer registries with low and high incidence rates for selected cancers for which the etiology is not well understood.

		Low incidence region	ASR*	High incidence region	ASR
**Prostate (C61)**		Thailand, Khon Kaen	3.1	US, Delaware: Black	206,7
**Gallbladder (C23–24)**	**Men**	UK, Wales	0.9	Chile, Biobio Province	11,3
	**Women**	UK, Wales	1.0	Chile, Valdivia	25,1
**Testis**	**Men**	Republic of Korea	0,6	Chile, Valdivia	13,7
**NHL (C82–85,C96)**	**Men**	India, Poona	3,5	US, California, San Francisco Bay Area: Nonhispanic White	18,8
	**Women**	India, Poona	1.9	Israel: Jews	14,4
**Kidney (C64)**	**Men**	Thailand, Bangkok	1.5	Czech Republic	22.1
	**Women**	Thailand, Bangkok	0,7	Czech Republic	9.9
**Pancreas (C25)**	**Men**	India, Chennai	1.6	Slovakia	11.2
	**Women**	India, Chennai	1.0	US, Detroit: Black	10.4
**Colorectal (C18–20)**	**Men**	India, New Delhi	4.9	US, Alaska: American Indian	64.8
	**Women**	India, New Delhi	3.3	New Zealand	36.0

* ASR = age standardized rate. ASR for low incidence regions based on at least 100 cases. (http://ci5.iarc.fr/CI5I-X/Default.aspx)

## How Can Genomics Fill These Gaps?

### DNA signatures of past exposures

**Fig 1:**
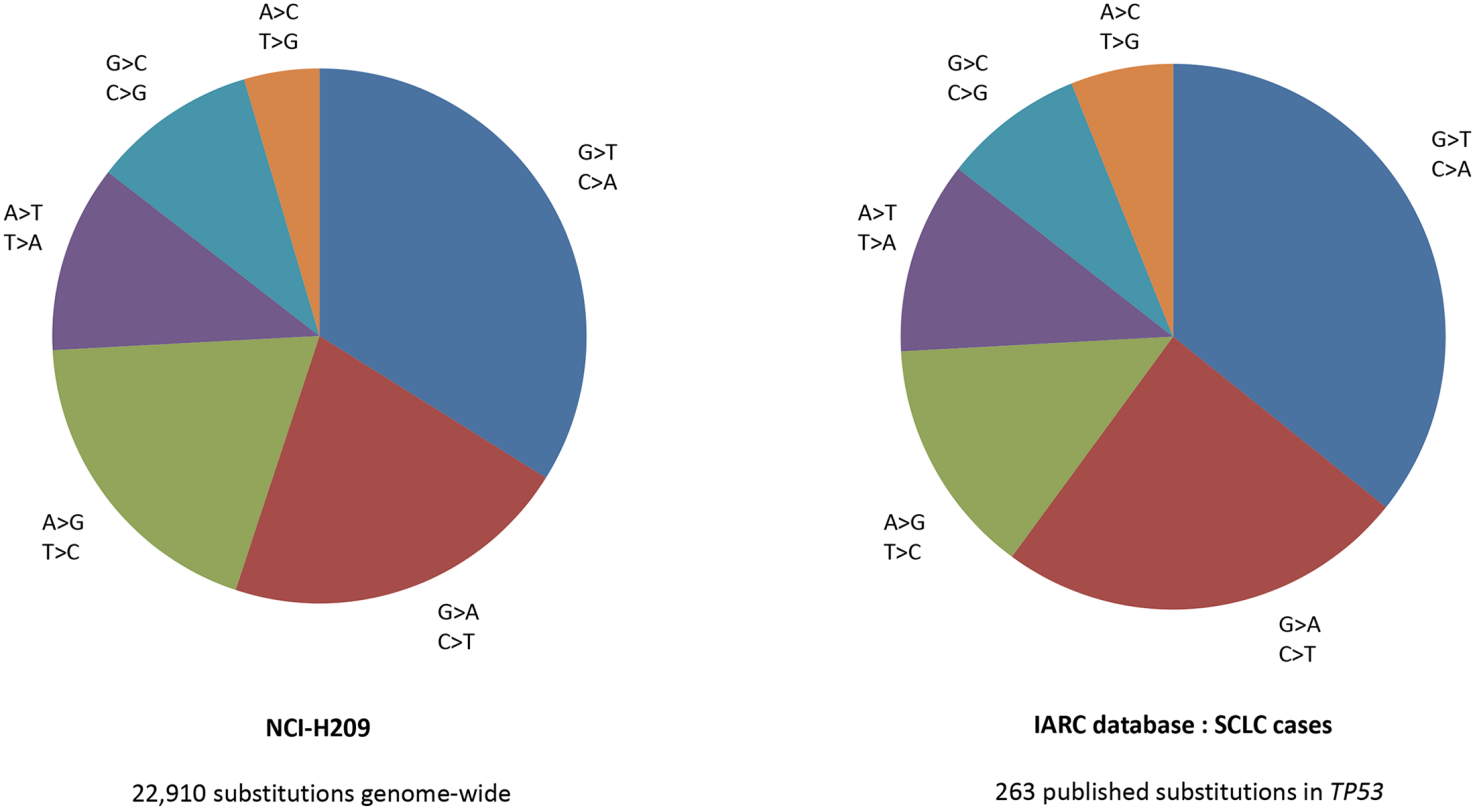
Comparison of (i) the distribution of 22,910 mutations identified from sequencing on SCLC line [19], with (ii) 263 published mutations from 253 SCLCs. IARC p53 database [18], accessed March 2015.

Certain environmental exposures can leave a mutation “signature” in the tumor [17], providing evidence on the specific lifestyle and environmental exposures that caused the tumor to occur. For example, patterns of mutations in the *TP53* gene, the most commonly mutated gene in cancer, differ strongly between smokers and nonsmokers who develop lung cancer, the former having a higher proportion of mutations that change a guanine base to a thymine (a G>T transversion). The changes are more likely to occur in a specific sequence context, with CpG dinucleotides being particularly enriched, and are more frequently found on the untranscribed DNA strand. The presence of such mutations can be explained by the direct mutagenic activity of specific compounds found in cigarette smoke, in particular polycyclic aromatic hydrocarbons (PAHs). The International Agency for Research on Cancer (IARC) P53 database has been documenting the pattern of p53 mutations within different cancers since 1989. Small cell lung cancers (SCLCs) occur almost exclusively among smokers, and among the 263 p53 mutations that have been recorded from 253 tumors, 32% are G>T transversions [18]. What is surprising is that the pattern of mutations across the whole genome of a single SCLC tumor is almost exactly the same as in the TP53 gene across 263 SCLC tumors. In 2009, a whole genome sequence of a SCLC identified 22,910 substitutions across the genome, with over one-third being G>T transversions [19] (Fig 1). While the vast majority of the mutations in any tumor have no functional impact, and are called “passenger mutations,” their pattern can be strongly indicative of the background exposures of the individual. Similar phenomenon can be observed for the pattern of mutations caused by sunlight and melanomas, or by aflatoxin B1 and liver cancer [20].

**Fig 2:**
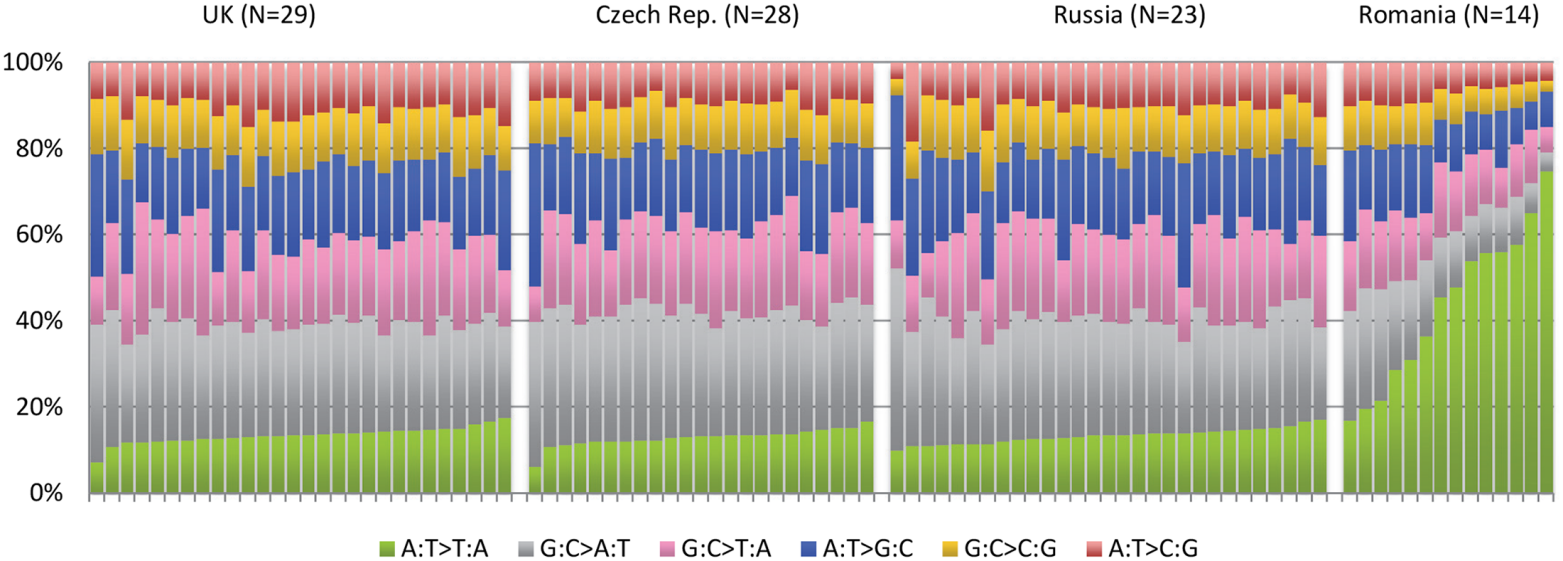
Mutation patterns from whole genome sequencing of 94 conventional renal carcinomas from four different countries showing a notable excess in the proportion of A>T mutations in cases from Romania. See Scelo et al. [21].

An example of how this type of investigation can expand the role of known carcinogens comes from the recently completed ICGC study of renal tumors [21]. Among 94 individuals with whole genome sequence data, recruited from four different countries, there was a sharp disparity among the pattern of A>T mutations among the 14 Romanian renal cancer cases when compared to the remaining 80 cases from the United Kingdom, Czech Republic, and Russia (Fig 2). A>T mutations are relatively rare in all tumor types, although many will occur as a result of exposure to aristolochic acid, a toxin that results from ingestion of the Aristolochia plant. Exposure is prevalent in parts of Asia, where it is common in traditional herbal remedies, and has been linked to rare cancers of the upper urinary tract that also exhibit a predominance of A>T mutations [20,22]. In the Balkan region of Southeastern Europe, the presence of Aristolochia clematitis (also known as European birthwort) is known to grow in wheat fields, contaminating the grain, and has been linked to Balkan endemic nephropathy, a renal disease that occurs in very specific regions along the Danube. The mutation profile in the 14 Romanian cases showed a predominance of A>T mutations on the untranscribed DNA strand and also occurred in a particular sequence context, two other facets of this signature that have been seen elsewhere. The results provide important clues that aristolochic acid exposure may be an important renal carcinogen in this part of Europe, going beyond the very specific region affected by Balkan endemic nephropathy.

### A catalogue of mutation signatures

In a comprehensive analysis of over 7,000 cancer cases with mutation data and close to 5 million mutations, Alexandrov and colleagues identified over 20 distinct mutation signatures, with most individual cancers showing evidence of more than one mutational signature [23]. Beyond those known to be caused by tobacco, UV light, and some specific alkylating agents, the cause of most of these signatures is not known or can only be hypothesized. While it cannot be expected that all of these mutation signatures will be linked to exogenous exposures, it is feasible that some will; and identifying the causes of specific mutation signatures and linking these to new cancer types will be an important step in expanding our knowledge on the causes of cancer. Evidence linking mutation signatures to specific exposures is likely to come about through two sources. One will be model systems whereby cell lines or other models are exposed to specific carcinogens and the resultant mutation profile is identified. For example, the human p53 knock-in (Hupki) mouse and derived immortalized mouse embryonic fibroblast models have been used to clarify mutation signatures for various exposures including UV light, benzo[a]pyrene, aristolochic acid, and aflatoxin B1 [24].

Another strategy to identify mutation signatures will be the comparison of sequence data from large numbers of cases included in studies such as ICGC and TCGA. An important characteristic of these studies up to now is that patients are recruited from single settings, with a strong focus on collecting high quality biological samples and accurate clinical data, but with only limited environmental or lifestyle data. An alternative approach that would maximize the possibility to find different mutation signatures would be to recruit an international series of cases that cover low and high incidence areas in a coordinated manner and also collect accurate information on lifestyle and environmental information. An additional strategy would be to select cases for sequencing based on the presence of a known or suspected carcinogen—the aim being to try to further define mutation signatures for an exposure. One could envisage a comparison of colorectal cancer tumors among individuals with high meat consumption compared to vegetarians, or a comparison of individuals with a history of heavy exposure to specific pesticides compared to no exposure for various cancers where this association has been hypothesized. While the large TCGA and ICGC sequencing initiatives were not established to identify lifestyle and environmental causes of cancer, there is an important opportunity to incorporate this aim in any future international initiative. This will require a commitment by those leading such studies to include key exposure data relevant to the cancer being studied.

### Germline variation and Mendelian randomization

An important limitation of the branch of epidemiology research that seeks to identify causes is that it relies on observations, and lifestyle characteristics of individuals inevitably correlate, resulting in the potential for confounding. For example, heavy alcohol drinkers will have higher rates of lung cancer simply due to their increased propensity to smoke. Any causal role for alcohol is thought to be unlikely [25]. Less straightforward are epidemiological findings that highlight a strong association between a particular cancer and some nutrients or foods. For example, one of the reasons that a protective effect was hypothesized between lung cancer and beta carotene was because of the consistent results from observational studies showing a protective effect for specific food types rich in this compound [26]. Subsequent randomized trials to test this hypothesis proved negative and, if anything, found an association in the opposite direction [27]. While epidemiologists frequently try to untangle these disease–exposure relationships, it requires a complete knowledge of how exposures correlate and the ability to control for them or measure them accurately, something that is rarely the case. Unmeasured or poorly adjusted confounders are one of the primary reasons for why epidemiological studies are unable to investigate important exposure–disease relationships or even get the wrong answer [28].

**Fig 3:**
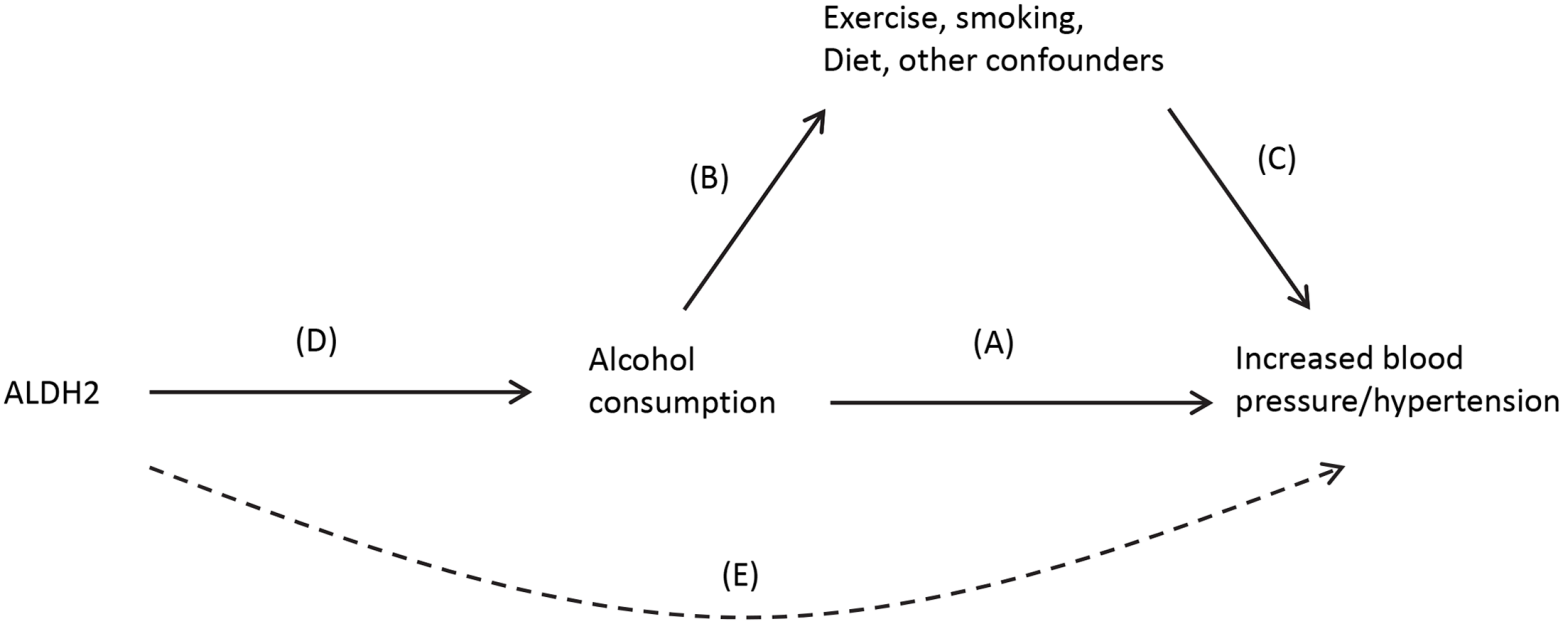
Causal pathway indicating how ALDH2 is an unconfounded marker (or instrument) of alcohol consumption in the association between alcohol and blood pressure.

An attractive option that can mitigate these shortcomings is Mendelian randomization [29]. This involves identifying a gene (or panel of genes) that is associated with the exposure and using this as an unconfounded “instrument” of the exposure instead of the exposure itself. It was Mendel who first recognized that genetic variation encapsulates information on physical attributes and that the information on different genes tends not to correlate (his Law of Independent Assortment). He also hypothesized that alleles are inherited in a random fashion from one generation to the next (his Law of Segregation). While these statements need certain clarifications, e.g., genes in close vicinity are likely to be inherited together, and Mendel did not use a terminology that included “genes” or “alleles”; in practical terms, this means that the selection of which individuals within a population who are more likely to have a genetic trait for smoking, drinking, or obesity is largely random, and these characteristics are inherited independent of other possible confounding factors. As an illustration, observational studies point to an association between alcohol consumption and increased blood pressure (arrow A), although there is much potential for confounding from lifestyle and socioeconomic risk factors that are also associated with both alcohol (arrow B) and hypertension, independently (arrow C) (Fig 3). The causality of the relationship (A) is unclear, as it may occur as a result of (B) and (C), and randomized studies are not feasible [30]. A Mendelian randomization analysis can utilize the *ALDH2* gene as an unconfounded indicator of alcohol consumption (D). *ALDH2* encodes for an enzyme that transforms acetaldehyde to acetic acid, and individuals who are homozygous for the null variant (2/2) drink considerably less than those who are homozygous for the active variant (1/1) with heterozygous (1/2) carriers in between. Further, *ALDH2* genotype has been found to be not associated with other risk factors of blood pressure such as smoking, exercise, and obesity. *ALDH2* is thus an indicator of drinking behavior that is inherited in a random fashion within a population and not associated with common confounders. Any effect of *ALDH2* gene on blood pressure should be due simply to its effect on alcohol consumption patterns and be independent of potential confounders (i.e., [E], the dotted line in Fig 3). A combined analysis of multiple studies reported a clear association with the ALDH*1/1 genotype and both blood pressure levels and diagnosis of hypertension, providing persuasive evidence of a causal association [30].

The potential for Mendelian randomization is apparent from recent publications on genes that influence obesity and adenocarcinoma of the esophagus [31], high fasting insulin levels and endometrial cancer risk [32], vitamin D genes and both all-cause and cancer-specific mortality [33], and also for cardiovascular disease and lipid levels [34–36]. The evidence linking obesity with esophageal adenocarcinoma is important as it is unlikely to be confounded by other potential risk factors including physical activity or specific dietary patterns. Similarly, the evidence linking endometrial cancer risk with insulin levels is helping to highlight the complex yet potentially causal relationship between risk factors for diabetes and cancer. Mendelian randomization studies have been compared to randomized control trials, and while Mendelian randomization studies have many attractions, they also have important limitations. In particular, one needs a genetic indicator (or instrument) for the exposure of interest. These have not yet been identified for many exposures including most lifestyle related factors, nor for many specific vitamins or nutrients. Given that extensive genome-wide analysis (GWA) data from very large population cohorts is becoming available, such as in UK Biobank, better instruments for Mendelian randomization studies are certain to be identified [37]. Additional issues include the pleiotropic nature of genes and that the genetic effect on the outcome trait is generally modest, meaning that very large studies are required to have a sufficient power to test the association between gene and outcome [38–41].

## Secondary Prevention of Cancer—Catching It Early

There were about 8 million cancer deaths estimated to have occurred globally in 2012, compared to 14 million new cancer cases [42], providing a crude estimate that over half of cancer patients die from the disease worldwide. Even in countries classed as having a very high level of human development, the ratio of deaths to new cases is nearly one in two. Cancer survival in highly developed countries has shown some improvements over recent decades, although nothing like the improvements seen for cardiovascular disease [43]. While access to high quality coordinated treatment is a major driver in these improvements, detection of cancer at an early stage followed by access to high quality care are fundamental criteria in increasing the chances of surviving a cancer diagnosis. Cancers for which survival is particularly poor, for example lung, pancreatic, and liver, continue to be frequently identified at stage III or IV, even in highly developed countries. The overall cancer survival situation is far worse in less developed countries, where a majority of cancers is diagnosed at later stages, and access to effective cancer care is generally more limited.

Existing screening programs for cancer may be enhanced in the future by identification of individuals at increased genetic risk using combinations of rare high risk variants and multiple common yet low risk variants [44–46]. However, a major breakthrough for cancer prevention would be the identification of biomarkers for early cancer detection that are (i) easily measureable, (ii) sensitive for presymptomatic cancer (i.e., picking up a large proportion of cases), and (iii) specific (giving a negative response in the overwhelming majority of cases that do not have the disease). Some cancer biomarkers have been identified that are predictive of subsequent disease but lack the necessary accuracy for routine use in a population. One recent example of a highly specific, sensitive, and easily measurable biomarker linked to a preclinical cancer is antibodies to HPV16 E6 and cancer of the oropharynx [5]. This biomarker is detectable in the plasma of the majority of individuals who develop a HPV-associated oropharynx cancer up to 15 years before onset of clinical symptoms and is absent in over 99% of the comparable general population. The results using this biomarker are recent, and it is curious that it is not strongly predictive of other HPV-associated cancers such as cervix, although it does seem to predict a proportion of HPV positive anal cancers [47]. This biomarker is not currently ready as a screening tool primarily because even a specificity of 99% is too low for a very rare outcome such as oropharynx cancer, where the number of false positives would far outnumber true positives. The consequences of a positive HPV16 E6 result are also unclear, as precancerous lesions for oropharynx cancer have not been defined. While this may be seen as a proof of principle that sensitive and specific early detection cancer biomarkers can be developed, if a biomarker were to be identified for a common cancer with poor survival that had a similar sensitivity and specificity, the consequences for cancer prevention would be far reaching.

An emerging genomic technique that may prove key for the early detection of cancer is the analysis of circulating tumor DNA (ctDNA) in blood samples. The presence of cell-free DNA (cfDNA) in the blood has been recognized for many decades, with particularly high levels being observed in cancer patients [48]. A proportion of cfDNA in cancer patients is circulating tumor DNA (ctDNA) that is of much interest given its potential to act as a noninvasive biomarker for a malignancy. Indeed, ctDNA has been termed a “liquid biopsy” with potential applications including identifying response to treatment and relapse and even early-stage disease [49]. The proportion of ctDNA compared to the amount of cfDNA may be high (e.g., above 10%), especially for late-stage disease or for large tumors, while for early-stage disease this ratio is thought to be approximately 0.1%–1%. Extreme deep sequencing of 10,000 fold or more does appear capable of identifying such small concentrations, and a recent evaluation of 640 patients with various tumor types found that among early-stage cases that had not spread beyond the initial site, ctDNA could be detected in about 50% [49].

There are alternative techniques for detection of preclinical cancers that may prove to be more feasible than ctDNA in the long term and that are already providing exciting data. A good example is the analysis of panels of microRNAs (miRNAs) from plasma or serum samples. The number of human miRNAs is limited (around 1,000), and they have several attractive features as a biomarker, including their relative stability over time [50]. The most promising data is currently from within lung cancer screening studies, where several groups have independently reported panels of a modest number of miRNAs measured in blood samples taken prior to diagnosis that have good sensitivity and specificity for subsequent lung cancer risk [51,52]. Such analyses could be an important adjunct along with smoking history and other lung cancer risk markers when deciding who should undergo screening for lung cancer using low dose computed tomography. Early detection of cancer using ctDNA mutations or miRNA analysis represent just two possible strategies, with others including identification of circulating tumor cells, metabolomics or proteomics analysis, identification of aberrant immunology profiles, and also epigenetic analysis of circulating tumor DNA.

There are important challenges to identifying accurate biomarkers for cancer prior to clinical onset of symptoms. Difficulties include access to appropriate biological samples from population cohorts, lack of standard protocols for dealing with biological samples, sequencing analysis, and bioinformatics procedures. While impressive, these difficulties may not be insurmountable, and given the payback from the successful development of such techniques, it would seem important to consider how this line of research could be prioritized in a similar fashion to the coordinated international cancer sequencing programs that have proved so successful. This will involve establishing international partnerships of key laboratories, agreed goals for testing laboratory and bioinformatics protocols, and sharing of common sets of samples, and subsequently of data and results. The key ingredients to any successful program will be clear objectives, leadership, availability of large research infrastructures to test biomarkers, international willingness to collaborate, and financial backing.

## Summary

It is known that the cancer burden will rapidly increase over the next 15 years, with an estimated annual number of new cases in excess of 20 million by 2030 [53]. Much of this increase will occur in parts of the world where the health systems are least capable of absorbing such an increase. New generations of cancer treatments often promise only an extended remission from disease and not cure and place an important financial burden on health services. There is no country in the world that will be able to treat its way out of this cancer problem [54].

Cancer epidemiology in the late 20th and early 21st centuries has made important contributions to reducing the numbers of cancers that would otherwise occur. By combining with the revolutionary new tools of genomics it can be expected to continue to produce similar findings that will lead to breakthroughs in identification of new causes of cancer and early detection. There is a danger, however, that, in the absence of a coordinated strategy on an international level, this progress will occur in fits and starts, leading to delays, waste of resources, and missed opportunities.
